# Ligand binding and global adaptation of the GlnPQ substrate binding domain 2 revealed by molecular dynamics simulations

**DOI:** 10.1002/pro.3981

**Published:** 2020-11-03

**Authors:** Maximilian Kienlein, Martin Zacharias

**Affiliations:** ^1^ Physik‐Department T38 Technische Universität München Garching Germany

**Keywords:** advanced sampling, conformational dynamics and substrate binding, coupled domain motion and binding, free energy simulations, GlnPQ ligand binding, ligand‐induced global transition, molecular simulations, periplasmic transporter

## Abstract

Substrate‐binding domains (SBD) are important structural elements of substrate transporters mediating the transport of essential molecules across the cell membrane. The SBD2 domain of the glutamine (GLN) transporter from bacteria consists of two domains D1 and D2 that bind GLN in the space between the domains in a closed conformation. In the absence of ligand, SBD2 adopts an open conformation with increased domain distance. In molecular dynamics (MD) simulations in the absence of ligands, no closing of the open conformation was observed on the MD time scale. Addition of GLN resulted in several reversible binding and unbinding events of GLN at the binding site on the D1 domain but did not induce domain closing indicating that binding and global domain closing do not occur simultaneously. The SBD2 structure remained in a closed state when starting from the GLN‐bound closed crystal structure and opened quickly to reach the open state upon removal of the GLN ligand. Free energy simulations to induce opening to closing indicated a barrier for closing in the absence and presence of ligand and a significant penalty for closing without GLN in the binding pocket. Simulations of a Leu480Ala mutation also indicate that an interaction of a C‐terminal D1‐tail_471‐484_ with a D2‐helix_418‐427_ (not contacting the substrate‐binding region) plays a decisive role for controlling the barrier of conformational switching in the SBD2 protein. The results allow us to derive a model of the molecular mechanism of substrate binding to SBD2 and associated conformational changes.

## 1 INTRODUCTION

ATP‐binding cassette (ABC) transporters form a large superfamily of integral membrane proteins in all kingdoms of life and play a major role in the transport of essential molecules across the lipid cell membrane.^1^, ^2^, ^3^, ^4^ For example, ABC transporters are involved in the uptake of cellular building blocks like amino acids and nutrients or participate in the excretion of waste products to the external milieu.^5^, ^6^ ABC transporters are dimeric proteins that consists of two transmembrane domains (TMDs) and two cytoplasmic nucleotide‐binding domains (NBDs) as well as substrate‐binding domains (SBD). These SBDs mediate the initial binding of substrate and delivery to the translocation subunit.^5^, ^6^, ^7^, ^8^

**FIGURE 1:**
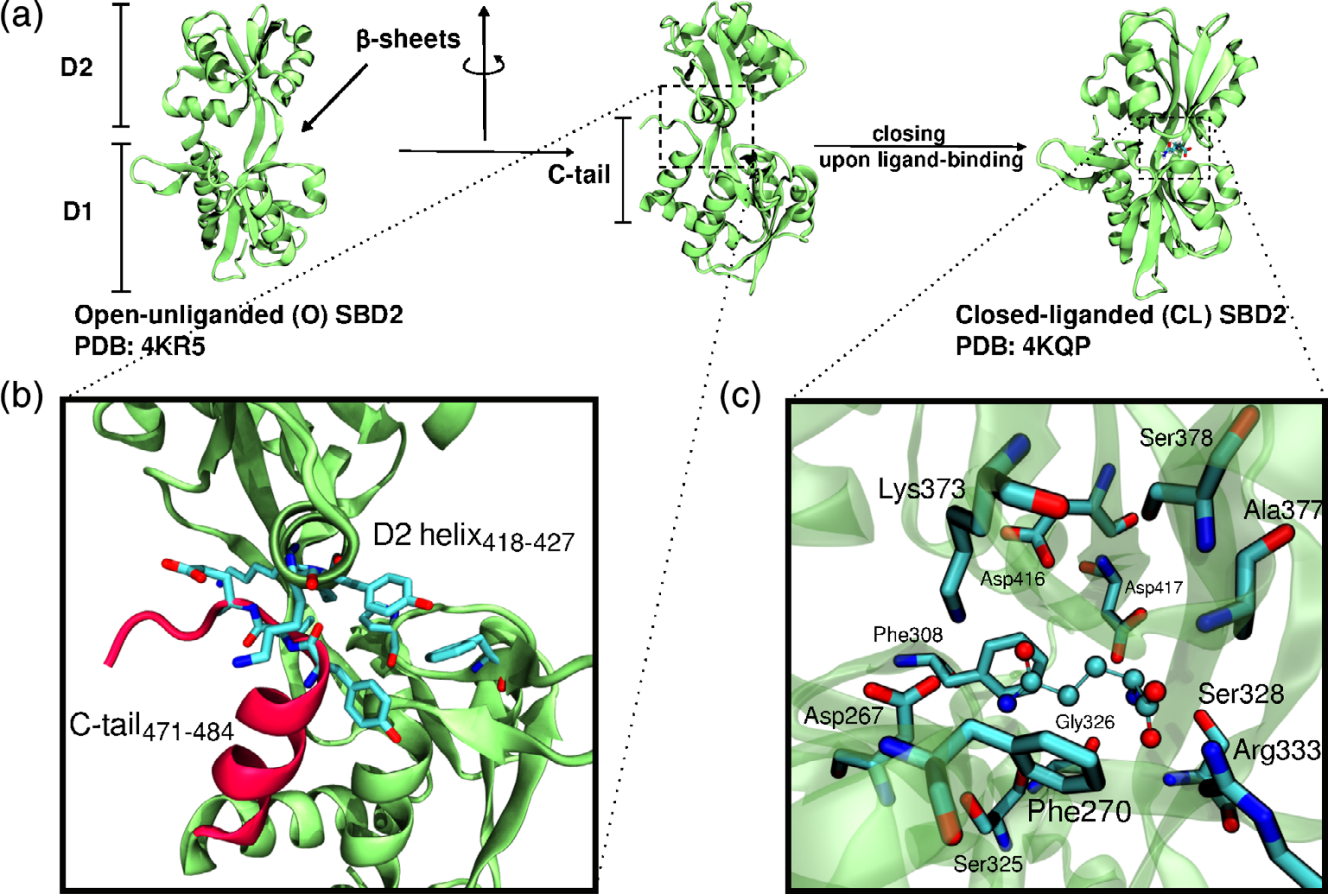
(a) X‐ray crystal structures of SBD2 of GlnPQ from *Lactococcus lactis* in open unliganded state and in the ligand (GLN)‐bound closed conformation. The SBD2 is composed of two continuous subdomains D1 (residues 255–343, 444–484) and D2 (residues 349–438) connected via two anti‐parallel β‐sheets (residues 344–348, 439–443). (b) The C‐terminal α‐ helical region (C‐tail: residues 471–484 in red, which are part of D1) interacts with D2 in the open‐protein state and contacts residues in a D2 α‐helix (residues 418–427). (c) The second inset panel illustrates the crystal structure contacts of GLN in the bound closed complex

Most of the SBDs are connected to the TMDs via a flexible linker.^7^, ^9^, ^10^ Three‐dimensional (3D) structures of many SBDs from various transporters have been determined in unbound (apo) as well as substrate‐bound (holo) states.^11^, ^12^ The SBDs consists typically of two domains (D1 and D2) with a ligand‐binding cavity in the space between the domains.^13^, ^14^ Without ligand usually an open domain arrangement is adopted. Crystal structures in the presence of substrate indicate a close domain–domain conformation around the bound substrate,^10^, ^12^, ^15^ and various studies indicate that the transition to a closed state is facilitated by ligand binding.^16^, ^17^, ^18^, ^19^, ^20^ After substrate binding the SBD complex binds to the translocator of the ABC transporter and delivers the bound substrate to initiate the translocation to the cytoplasmic side of the membrane. One of the most extensively investigated ABC transporters is the GlnPQ transporter from bacteria that uses two SBDs (SBD1 and SBD2) for the import of asparagine, glutamine and glutamate: The SBD2 binds specifically to L‐glutamine (GLN) and is essential for its subsequent transport.^19^ For SBD2 high‐resolution crystal structures of both the apo as well as holo form with bound GLN are available^10^ (Figure 1).

Nuclear magnetic resonance (NMR) coupled to paramagnetic relaxation enhancement has been used to characterize the open‐close transition^21^ indicating a dominance of the open state for the GLN binding protein in the absence of ligand. In contrast, the same technique suggested for maltose‐binding protein a minor population of a closed‐form even in the absence of ligand. In addition, extensive single‐molecule Förster resonance transfer (smFRET) experiments have been performed on SDB2 in the presence and absence of GLN to elucidate the dynamics of the ligand binding and domain closing processes.^6^, ^19^, ^20^, ^22^ In the smFRET technique the global domain–domain motion can be detected reaching a time resolution in the millisecond regime and has allowed to elucidate the detailed kinetics of the ligand‐binding and associated global structural change. It has been shown that the overall transport cycle of GlnPQ is highly influenced by the conformational dynamics of the SBDs.^20^ However, it is neither possible to detect individual substrate‐binding events if these do not trigger a global change nor to characterize short‐lived intermediate states. In addition, the detection of a smFRET signal may only allow one to distinguish different global structural changes qualitatively without further structural characterization on a molecular level.^23^, ^24^

We use molecular dynamics simulations on the SBD2 of the GlnPQ importer from the gram‐positive bacterium *Lactococcus lactis* to investigate the ligand binding process and associated conformational changes in atomic detail. This technique has already been used to study the dynamics of GLN binding proteins^25^, ^26^, ^27^ and related systems^28^, ^29^ mostly in the apo state to investigate local and global mobility. It can also be used to study how ligands change the protein’s internal energies and coupling to conformational changes.^30^ Starting from the open unbound structure the addition of GLN to the simulation box results in multiple reversible binding and unbinding events of the ligand to the larger of the two SDB2 domains (D1) forming basically the same contacts as in the known closed complex. Surprisingly, this does not trigger domain closing on the time scale of the MD simulations (several μs). Starting from the closed‐form without ligand results, however, in a transition to the open form. Using extensive free energy simulations, we characterize the free energy profile for the open/close domain transition in the absence and presence of bound substrate and find both a stabilization of the closed‐form and lowering of the transition barrier in the presence of the ligand. The open form is stabilized by a helical segment located near the hinge region between the domains that form non‐covalent contacts not present in the closed arrangement. The in silico substitution of a critical Leucine at the contact interface results in a rapid closing in the presence of the substrate demonstrating that this interaction is key to the conformational switching behavior of the system. The simulations allow us to derive a model for the molecular mechanism of substrate binding and associated conformational changes.

## 2 RESULTS AND DISCUSSION

### 2.1 *Simulation of the open SBD2 in the absence and presence of GLN*

**FIGURE 2:**
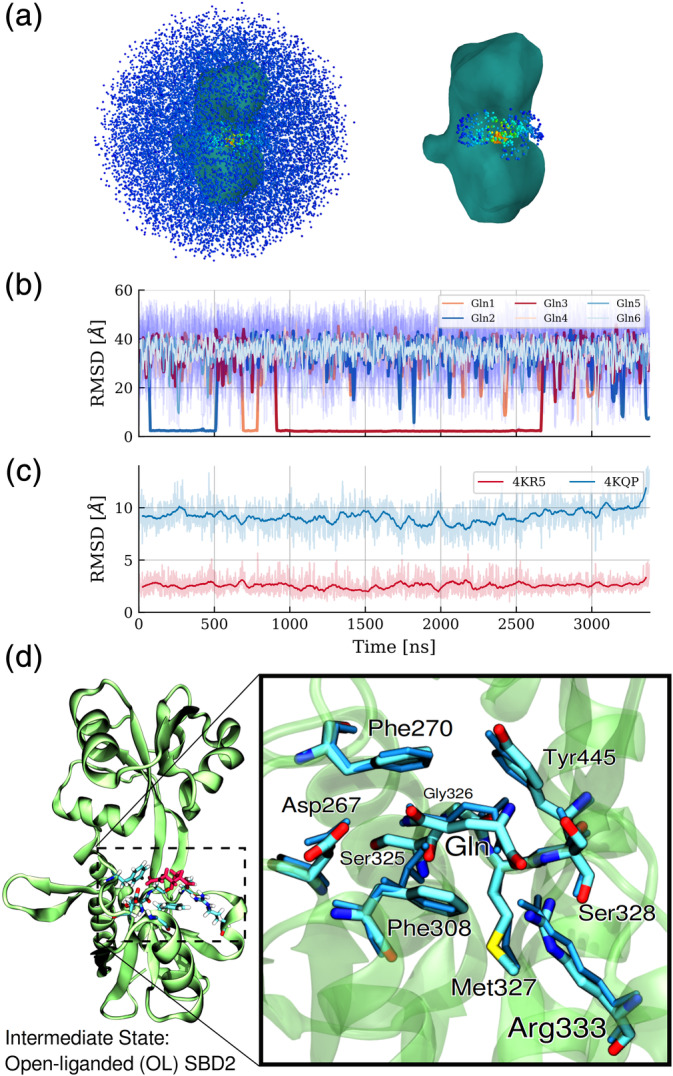
Ligand‐binding mechanism to the open‐protein form of SBD2. (a) The blue dots (left panel) around the SBD2 structure (shown as surface contour) indicate positions sampled by the GLN ligand during the simulation starting with the open SBD2 and six randomly positioned GLN molecules. In the right panel, only the sampling near the binding site on the larger subdomain D1 is illustrated (the color of each dot indicates the sampling density, with red representing a high density and blue low density). (b) Root‐mean‐square deviation (RMSD, original data, and running mean) of each GLN ligand (indicated as different line colors) from the native‐like binding position at the D1 domain (after best superposition of the D1 domain on the reference structure). (c) RMSD of the protein backbone during the MD‐simulation from the open (start) structure (pdb:4KR5) and from the closed state (pdb:4KQP). (d) Close‐up view of a snapshot with bound GLN at the binding site superimposed on the crystal structure (shown in blue stick representation). The α‐carboxyl group of L‐glutamine interacting with the guanidinium group of Arg333, forming a salt bridge, plays a major role in stabilizing this intermediate state

Unrestrained molecular dynamics (MD) simulations were started from the open SBD2 state (PDB: 4KR5). In the absence of GLN ligands the root‐mean‐square deviation (RMSD) with respect to the start structure stayed in the range of ~2–3 Å within 1.8 μs simulation time and no indication of spontaneous global domain closing. The RMSD with respect to the closed structure remained at a high level of ~9–12 Å (Figure S1). In a second simulation starting from the same SDB2 structure the simulation box contained six randomly placed GLN ligands. During the 3.3 μs simulation the GLN sampled extensively the surface of the open protein (Figure 2a,b). The GLN molecules stayed only a few nanoseconds at various surface positions (indicated by the large and rapidly fluctuating RMSD of most of the GLN molecules, Figure 2b). However, several binding events to the binding pocket very close to the geometry found in the crystal structure of the closed ligand‐bound form of SBD2 (PDB: 4KQP) were captured. Remarkably, binding to the open form occurred only to the binding region on the large subdomain (D1: residues 255–343 and 444–484) not contacting residues of the D2 (residues 349–438) domain.

The lifetime of these complexes was much longer (ranging from 100 to 1000 ns) than for the nonspecific placements distributed over the whole surface of the protein. After dissociation of one GLN, replacement with another GLN was observed two times indicating independent and uncorrelated binding events.

All the three captured complex formations resemble the ligand position in the crystallized placement and structure with RMSD_lig_ < 2.5 Å (the RMSD of the ligand heavy atoms after best superposition of the receptor on the experimental reference structure, Figure 2b,d). Surprisingly, on the time scale of the MD simulations

no domain closing transition was observed. Very similar to the simulation in the absence of a ligand the RMSD of the sampled states remain small relative to the open start structure and much larger relative to the closed experimental structure (Figure 2c). In addition, no other significant change in the RMSD of the protein upon ligand binding was observed. Hence, the GLN‐binding to the specific binding‐site on the larger D1 domain of SBD2 does not induce a closing of the two domains to directly form the closed bound structure. This indicates a significant energy barrier for closing that is not crossed on our simulation time scale.

Since the simulation indicates a new potential intermediate SBD2 complexed state adopted prior to the domain‐closing event, it is of interest to characterize the binding process and how it is stabilized in detail. For the time intervals with a site‐specifically bound GLN we performed an MMPBSA trajectory analysis to estimate the mean interaction contributions of individual residues in the SBD2 to stabilize GLN binding (Table 1).

**TABLE 1:** Per residue interaction energy decomposition of the open SBD2‐GLN complexed state

Residue	ΔG (kcal/mol)
Arg333	−6.3 ± 0.8
Gly326	−2.3 ± 0.4
Ser325	−1.7 ± 0.5
Asp267	−0.3 ± 0.5
Ser328	−2. 6 ± 1.0
Met373	−2.2 ± 0.4
Phe270	−2.1 ± 0.6
Phe308	−2.5 ± 0.5

*Note:* Interaction energy contributions are calculated with the MM‐PBSA method using the structures generated by the MD simulation of the open SBD2 structure for time frames with a GLN bound to the SBD2‐D1 domain.

The calculated total MMPBSA binding energy was −9.4 kcal/mol (including a quasi‐harmonic entropy term). The analysis of individual contributions revealed Arg333 to play the dominant role in stabilizing GLN binding by a salt bridge of the terminal COO− through the guanidinium group of Arg333. The backbone carbonyl of Gly326 as well as the hydroxyl group of Ser328 also contribute significantly to ligand binding by forming frequent hydrogen bonds to the GLN alpha‐amino group. The bound GLN side chain amino group forms stabilizing hydrogen bonds with Asp267 or Ser325 as well as non‐polar interactions with the two opposing benzyl groups of Phe270 and Phe308.

**FIGURE 3:**
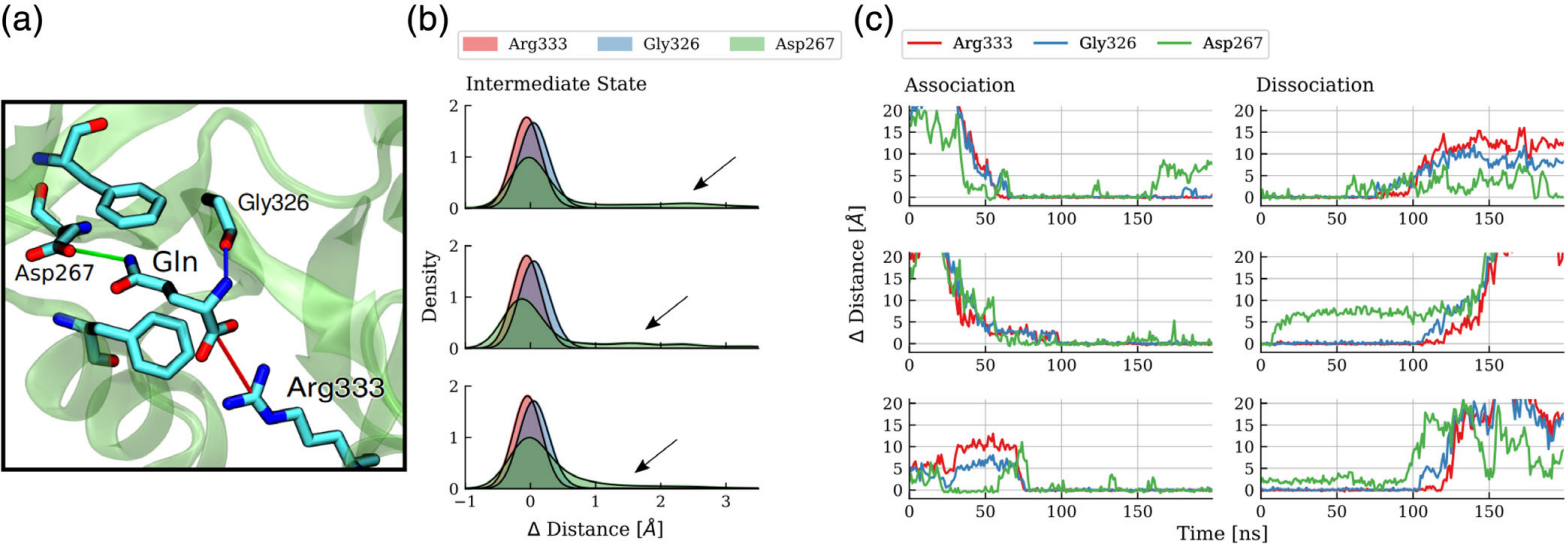
Characterization of GLN ligand association and dissociation (with SDB2‐D1) at high time resolution. (a) The overall position and orientation of GLN in the binding site on the D1 domain can be characterized by three contact distances in the ligand‐bound crystal structure: The distance between Arg333:CZ and GLN:C atom (red line), Gly326:O and GLN:N atom (blue line) and Asp267:CG and GLN:NE atom (green line). The sampled distance‐differences with respect to these reference distances in the crystal structure were used to capture the binding dynamics of GLN. (b, c) Sampled distance distributions and time resolved distance sampling for three association and three dissociation events of GLN during the MD simulation. In the intermediate state (liganded, but open protein form) GLN's amino group occasionally loses contact to the binding pocket (Asp267, Ser325) leaving the ligand solely stabilized by its charged functional groups and the hydrophobic contacts (high distance differences for Asp267: black arrows). In addition, when examining the association/dissociation for the binding events, especially for the dissociation the contact to the Arg333 is the one firmly stabilizing GLN

Assuming sufficient sampling of the underlying thermodynamic ensemble via this extended free MD simulation we determined the overall free energy difference of GLN binding in a straightforward fashion, by directly counting the fraction of simulation time spent in the respective states. The total simulation time amounted to 3,384 ns in which GLN was bound to SBD2 for 2,270 ns, leading to probabilities of P_bound_ = 0.67 and P_unbound_ = 0.33. With a ligand concentration of C_ligand_ = 25 mM a standard binding free energy of GLN binding of ΔG = −2.6 kcal/mol was obtained. To further analyze the dynamics and intermediates of the ligand binding process we investigated each association and dissociation event at higher time resolution. We recorded three distances in distinct regions of the binding site that characterize the bound state (Figure 3). No distinct order of contact formation for the different events was observed. In intermediate states the GLN contacts to Arg333 and Gly326 are sharply peaked around the placements found also in the crystal structure of the bound complex and the formation as well as disruption occurs almost simultaneously. In contrast, the distance of the substrate to Asp267 shows a broader distribution with occasional higher deviations from the arrangement in the crystal structure. However, in some binding events it is the first contact formed and also the first that is disrupted upon dissociation.

### 2.2 *Molecular dynamics simulations starting from the closed SBD2 structure*

**FIGURE 4:**
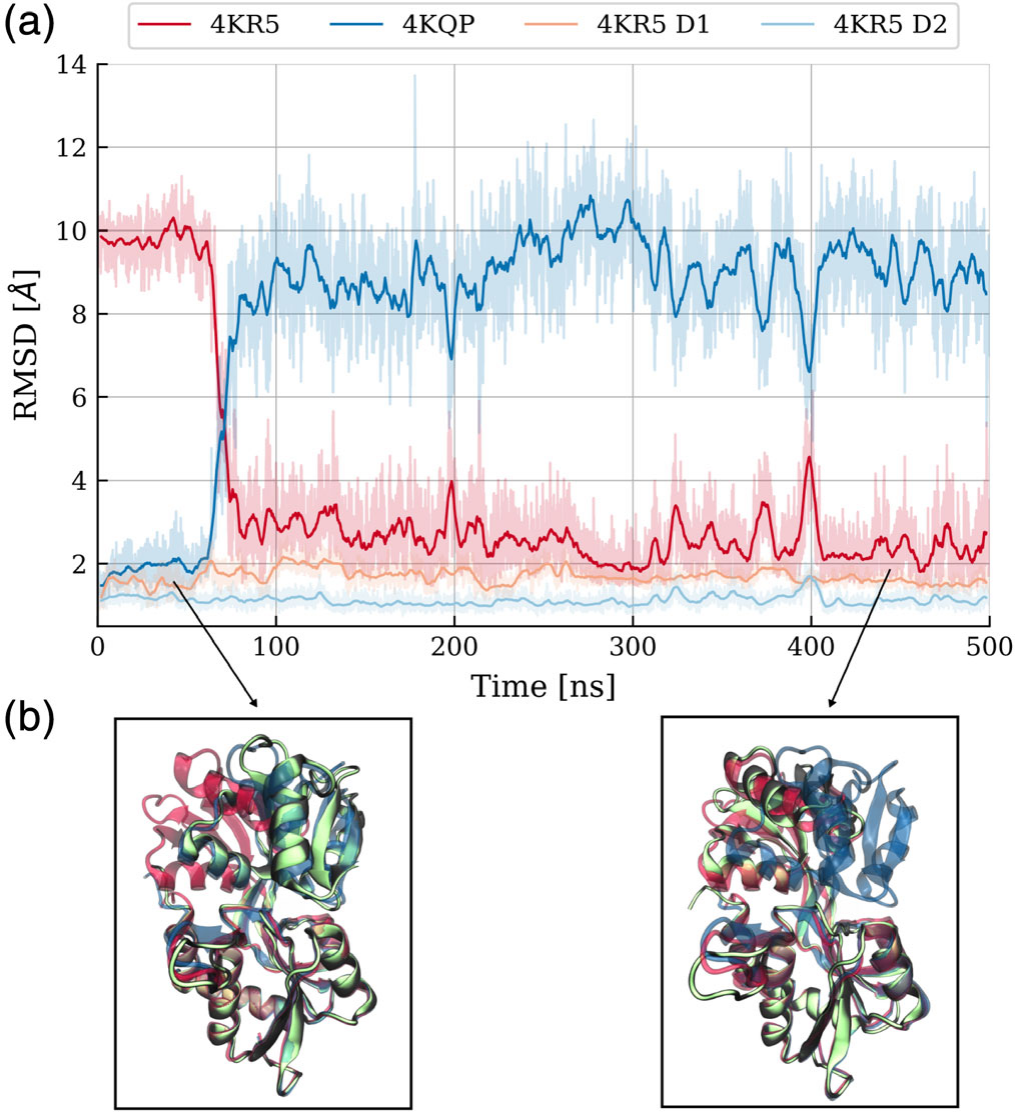
Large‐scale opening transition from the closed to the open conformation in the absence of ligand. (a) Cα‐ RMSD with respect to the crystal structures of the closed (PDB:4KQP, shown in blue) and open (PDB:4KR5, shown in red) conformations. The RMSD of the individual domains D1 and D2 from the corresponding segments in the experimental start structure are also shown (yellow and light‐blue lines, respectively). (b) Superposition of snapshots (with respect to D1 domain) during the simulation (green cartoon) onto the closed native structure (blue) and open native structure (red)

**FIGURE 5:**
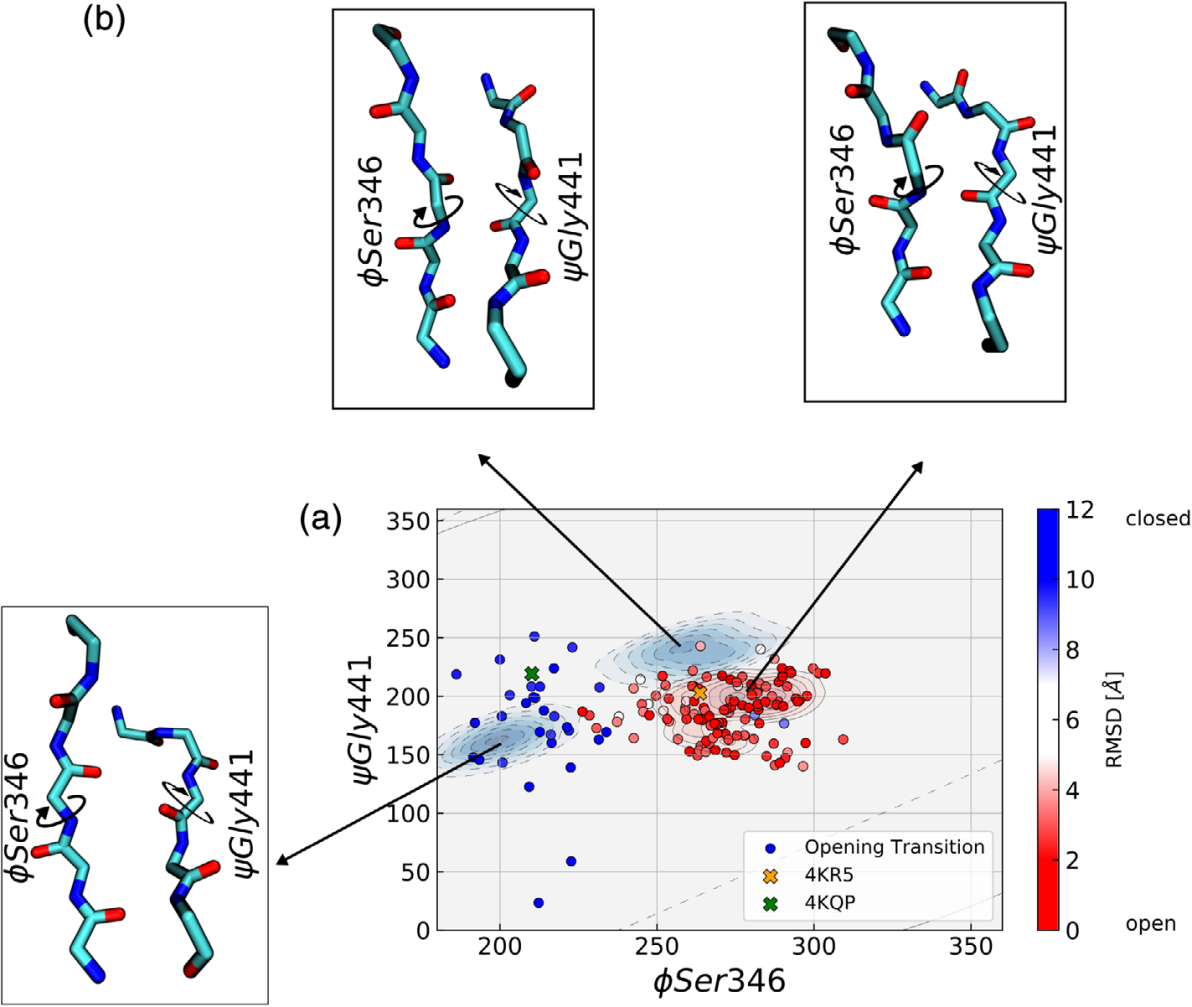
Conformational changes in the β‐sheet hinge region upon opening transition of SBD2. (a) The Φ‐Ser346 and ψ‐Gly441 dihedral angle distributions are depicted as blue and red contours extracted from the simulations of the open (red) and closed (blue) SBD2 structures. In addition, the states sampled during the transition from a closed to an open conformation (observed in the time interval of 60–90 ns as illustrated in Figure 4) are indicated by blue to red dots (color scaled by deviation from closed vs. open SBD2 structure). The reference dihedral angles of the respective crystal structures are shown as crosses (orange, green). (b) Conformational snapshots of the hinge region that correspond to the dominant states are illustrated as stick models

Simulations starting from the closed conformation (PDB: 4KQP) with GLN in the binding cleft between D1 and D2 domains indicated stable binding and also no tendency for global opening on the time scale of 1 μs (Figure S2). However, the removal of the ligand (in silico replacement and solvation) from the closed crystal structure appears to destabilize the closed domain arrangement of SBD2 (Figure 4). During the simulations the closed state opens up within less than 100 ns and adopts a global open conformation in close agreement with the experimentally observed apo structure (Figure 4). The structures of the subdomains D1 and D2 do not change significantly upon opening with the RMSD not exceeding 2–3 Å (similar to the observation when starting directly from the open SBD2, see above). The most significant local structural rearrangements take place in the β‐sheet hinge region. Backbone dihedrals in this region showed small fluctuations but are mostly uncorrelated with respect to the closed or open SBD2 structure. However, we identified two correlated dihedral angles, the Φ‐angle of Ser346 and the ψ‐angle of Gly441, that allowed a separation between open and closed SBD2 (Figure 5, S3). It appears that a correlated dihedral change of just these two backbone dihedral angles mediates the global domain rearrangement (of course, more subtle changes of other dihedral changes may also contribute).

**FIGURE 6:**
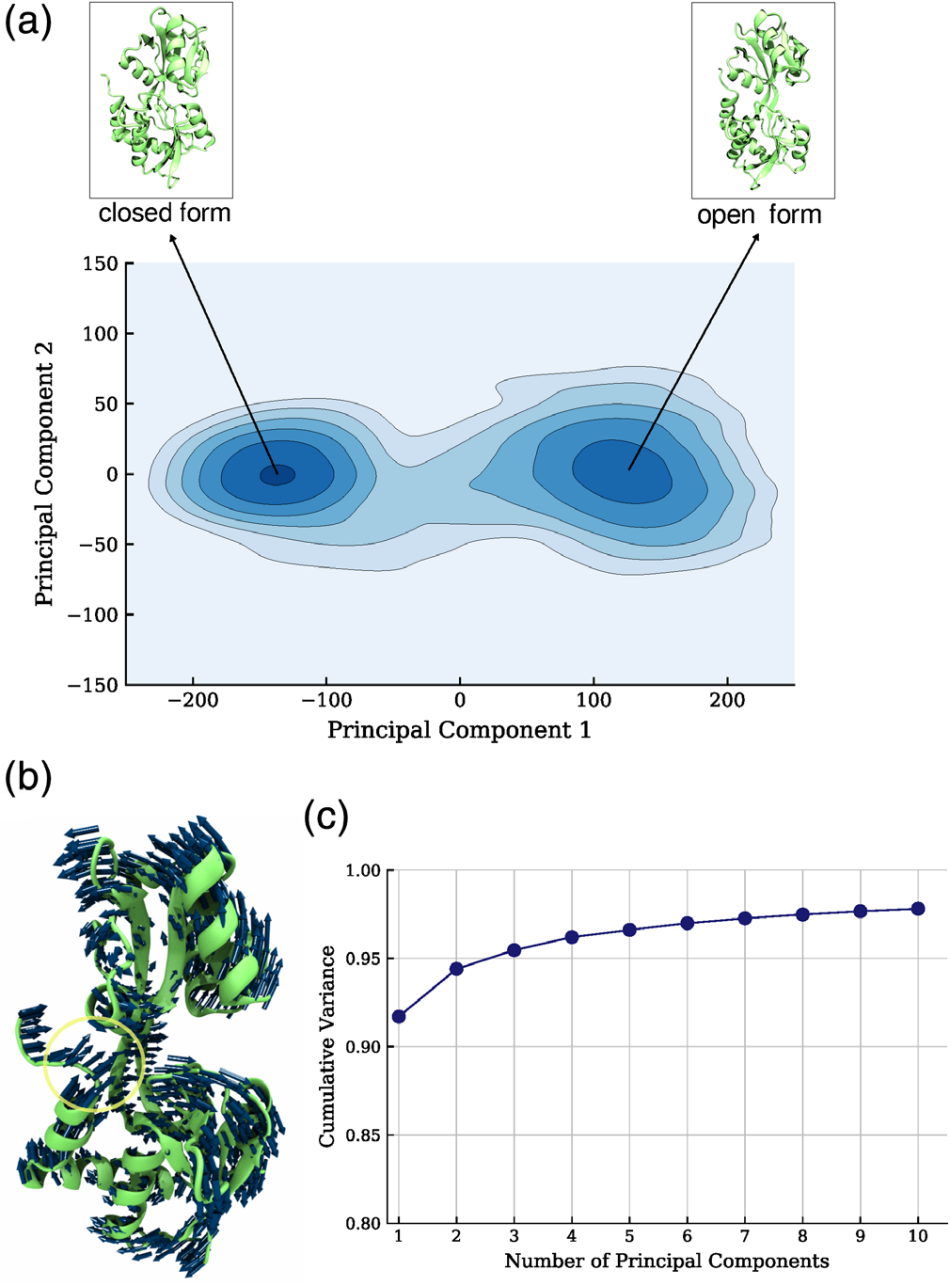
Principal Component Analysis of the opening motion, upon in silico removal of Gln. (a) Projection of the sampled transition onto the first two eigenvectors. (b) Illustration of the collective backbone atom directions of the first dominant PC (contributing ~92% of the variance). Note, that in the first component the transition of the two subdomains away from each other is correlated with a second collective movement of the D1 C‐terminal_417‐484_ region toward D2's α‐helix_418‐427_. (c) Cumulative contribution of the first 10 PCs to the total Cartesian variance sampled during the simulations

As a next step, we applied principal component analysis (PCA) to identify the correlation in the backbone dynamics of this opening transition and projected the trajectory onto the first two eigenvectors (Figure 6, S4). That accounts for almost 95% of the observed global conformational change (Figure 6). The covariance matrix was calculated with respect to the non‐hydrogen backbone atoms CA, C, O, and N. The main feature of the transition is the ordered shift of the two domains D1, D2 toward the open state and the PC1 direction also correlates well with the center of mass‐ distance between D1 and D2 (Figure 6b). Note, that the C‐terminal residues move toward D2's helix_418‐427_, especially in the realm of the key residue Leu480, which we will discuss in more detail subsequently (Figure 6b).

(comment: in Figure 6 the "old" numbering with capital letters (C) is still slightly visible…)

**FIGURE 7:**
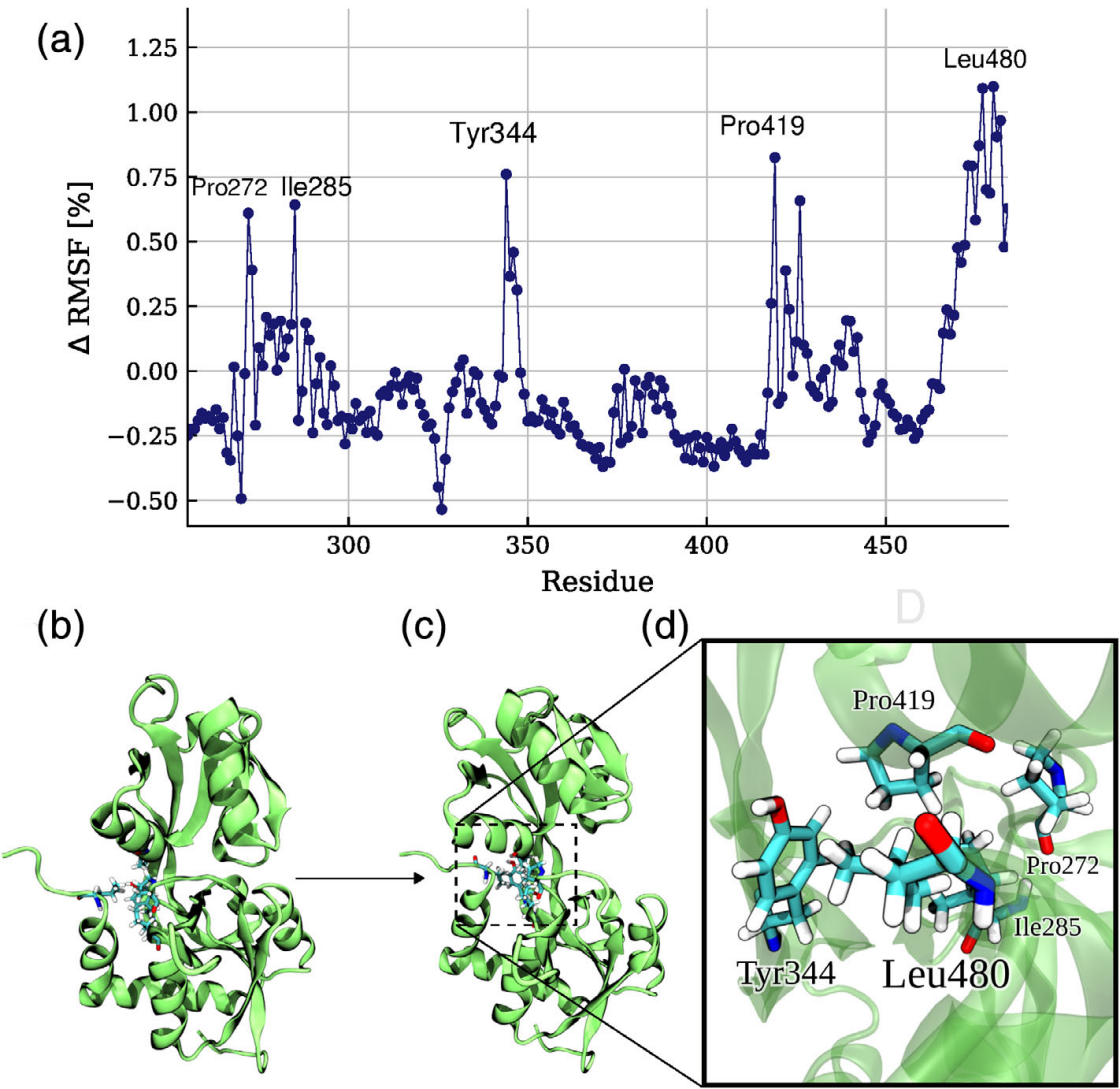
(a) Relative ΔRMSF per residue in SDB2 upon opening of the protein. A positive/negative ΔRMSF indicates a higher mobility in the closed/open form, respectively. The few residues with positive ΔRMSF indicate residues that participate in the D1‐helix_471‐484_ –D2‐helix_418‐427_ interaction. (b, c) Illustration of the formation of D1‐helix_471‐484_ –D2‐helix_418‐427_ contacts upon SDB2 opening. (d) A close‐up view of the interacting residues

The relative changes in root‐mean‐square‐fluctuation (RMSF) of each residue gives a measure of residues that showed different mobility in the two domain arrangements (Figure 7). Typically, ligand binding to a receptor leads to a reduction of conformational fluctuations and we also observe that the mobility of most residues is slightly smaller in the closed versus open states. Surprisingly, for several mostly hydrophobic residues the mobility is dramatically reduced in the open state compared with the closed form of SDB2 (Figure 7a). One can interpret this as an indicator of stabilizing interactions, especially visible for the C‐terminal (and D2 α‐helical_418‐427_) region when the protein is in an open state. All the residues with reduced mobility in the absence of GLN (Pro272, Ile285, Tyr344, Pro419, and Leu480) contribute to the formation of a hydrophobic “lock,” that stabilizes the open state of SBD2 (Figure 7b–d). Note, the spontaneously formed arrangement (not seen in the closed structure) observed during the unrestrained simulations starting from the closed form is in close agreement with the local arrangement found in the experimental structure of the open form (Figure S5).

### 2.3 *Effect of a point mutation Leu480Ala on the dynamics of SBD2*

The above‐unrestrained simulations starting from the closed SBD2 structure without ligand indicate that the C‐tail_471‐484_ residues of D1 form an interaction with D2's α‐helical_418‐427_ residues, to serve as a “lock,” stabilizing the open conformation. Despite the fact that other D1 residues (Pro272, Ile285) as well as tyrosine (Tyr344) in the β‐sheet hinge region also participate in this hydrophobic “locking” mechanism it is this close contact between the two helices which seems to be crucial in the open SBD2 state. Interestingly, this type of D1/D2 contact has been found in several SBDs (Table S1) and is characteristic for the SBD2 class of ligand binding proteins.

**FIGURE 8:**
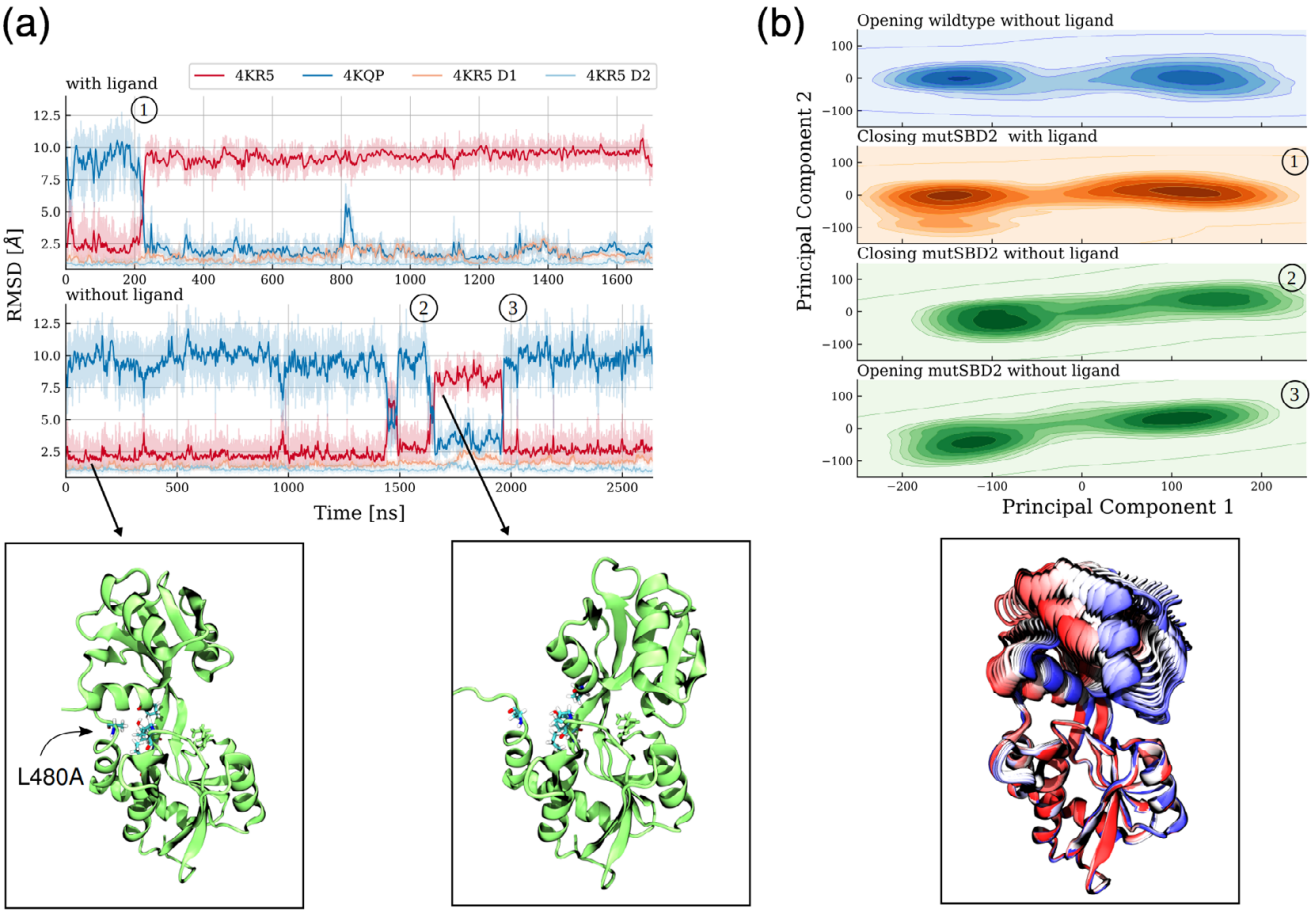
SBD2‐L480A reduces the open‐close transition barrier. (a) RMSD of sampled states from the native open (red) and closed (blue) states of SDB2 during simulations. The RMSD of individual domains D1 and D2 relative to the native structures is illustrated by yellow and light blue lines. Note, that the presence of the ligand (upper panel) in (a) drastically increases the lifetime of the closed state. Typical sampled snapshots of the open and closed state are shown as cartoons below the plot. (b) Projection of the sampled states from the SBD2‐L480A transitions onto the first two PC eigenvectors of the opening trajectory for the wild type protein. The height of the density is scaled logarithmically. The two sampled clusters represent the two stable protein states. All transitions followed the same pattern, namely a clear tilting motion. Multiple frames of a typical transition trajectory (lower panel in b) illustrate this tilting motion (shown in cartoons, colored from beginning [red] to end of transition [blue])

Especially, the Leu480 residue is centrally located at the hydrophobic pocket formed by residues of the D1 domain (Figure 7d) and shows also a much lower local mobility in the open vs. closed SBD2 structure (Figure 7a). The substitution of this residue by alanine is expected to lower the interaction between the two lobes of SBD2 and should reduce the energy barrier/penalty for global domain rearrangements. Hence, comparative MD simulations were started from the open structure of the SBD2 with the Leu480Ala in silico substitution either in the presence of a GLN ligand bound to the D1 domain (same geometry as observed in our simulations) or without a ligand (Figure 8).

Already after 220 ns of unrestrained MD simulation with GLN present the SBD2 undergoes a global transition from the open state toward the closed D1/D2 geometry with an RMSD of <2.5 Å with respect to the experimental closed structure (compared with no closing event within >3,000 ns for the wild‐type complex, Figure 2).

During this transition the conformation of the two individual D1, D2 subdomains remains close to the conformation in the crystal structure with an average RMSDs values of ~3 Å for D1 and 2.9 Å for D2. Again, the closing motion is controlled by backbone dihedral angle changes in the β‐sheet hinge region connecting the two lobes, with correlated changes in Φ‐Ser346 and ψ‐Gly441 separating the two states (Figure S3). After closing, the simulated structure remains close to the experimental crystal structure (Figure 8).

Interestingly, even in the absence of a ligand, a spontaneous transition to the closed‐form was observed (at around 1,650 ns). This transition also resulted in the disruption of the contact between C‐tail_417‐484_ and the α‐helix_418‐427_ of D2. However, the closed‐form showed larger conformational fluctuations than in the presence of a bound ligand (Figure 8a) and the lifetime of the state was only ~350 ns before it returned to the open SBD2 arrangement. All discussed transitions from open to closed protein conformation and vice versa seemed to be a clean tilting motion, be it with or without ligand. To verify this, we projected all the transitions for SBD2/Leu480Ala onto the first two eigenvectors for the wild type closed‐to‐open transition (scaling the density logarithmically, Figure 8a). The first eigenvector represents the global motion, clearly separating the two states of the protein. All transitions displayed a clear tilting motion, with disruption of the D1/D2 contact (lower panel Figure 8b).

### 2.4 *Free energy advanced sampling simulations of SBD2 domain motions*

The unrestrained MD simulations give important qualitative insights into the substrate dependent local and global dynamics of the SBD2 system. However, important transitions such as the open‐to‐close transition of the SBD2 protein upon ligand binding was only observed for a Leu480Ala mutation but not for the wild type indicating a substantial energy barrier not crossed within the simulated time scales. In order to investigate the free energy profile for open‐close domain transitions we performed extensive HREUS simulations on the system employing a center‐of‐mass distance reaction coordinate between the two subdomains D1 and D2 that allows to distinguish the open and closed state and correlated with the PC1 of the observed transition.

**FIGURE 9:**
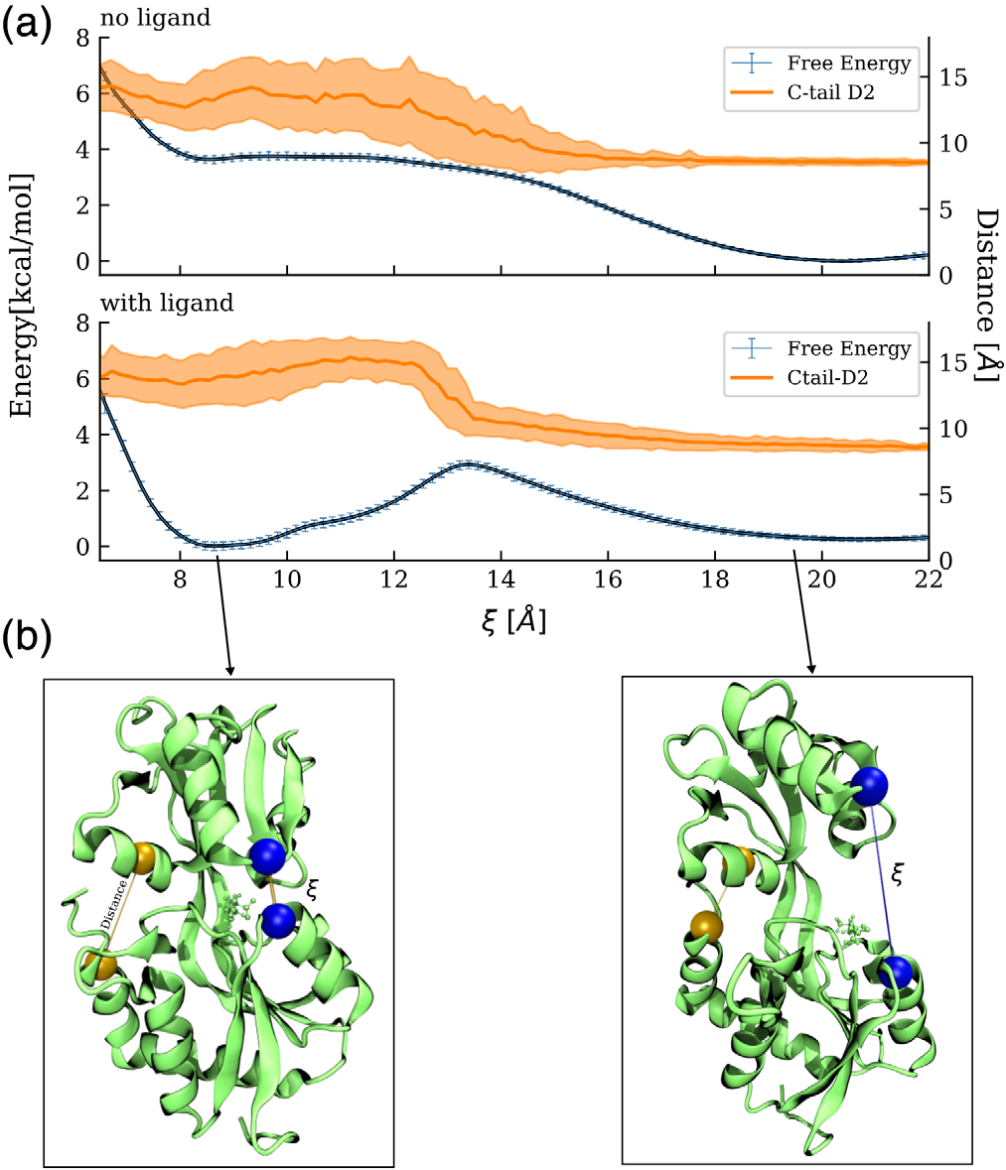
(a) Computed potentials of mean force (PMF) for the wild‐type SBD2 versus a center‐of‐mass distance coordinate between centers indicated as blue spheres in the protein structures (b) (see also Methods and Figure S6). The regime around ξ = 8.5 Å represents the closed state and around 19–20 Å the open SDB2 state. The presence of L‐glutamine in the binding site (lower PMF panel) stabilizes the closed state. In addition, the distance of the C‐terminal D1‐helix_471‐484_–D2‐helix_418‐427_ is illustrated. A small distance and small fluctuations indicates a stable contact with little fluctuations (in the open state) and larger distances and stronger fluctuations are observed for the regime representing the closed state. The mean values of this distance together with the standard deviation are shown

The HREUS free energy simulations were performed in the presence and absence of GLN in the binding cleft on the D1 domain. The frequent exchanges between Umbrella sampling windows in the HREUS approach result in good convergence of the calculated free energy profiles (Figure 9) but still requiring extensive sampling of ~500 ns per US interval (aggregate US simulation >30 μs). In the apo state the open domain arrangement is strongly favored by a free energy difference of ~3.9 kcal/mol. Only a small energy barrier of ~0.1–0.2 kcal/mol between closed state and transition toward opening was observed. In parallel to the induced closing or opening the C‐tail_471‐484_‐D2 helix_418‐427_ contact is formed/disrupted (Figure 9b). In the presence of a GLN ligand in the binding pocket the free energy of the closed state is found to be slightly lower (by about ~ −0.3 kcal/mol) compared with the open state. The barrier for open‐to‐close transition is similar to the barrier in the opposite direction and amounts to ~2.9 kcal/mol (Figure 9). Interestingly, the formation/disruption of the additional C‐tail_471‐484_‐D2 helix_418‐427_ contact coincides with the transition barrier indicating that this is the key‐element for the observed conformational switching and distinction of two stable states of the system.

**FIGURE 10:**
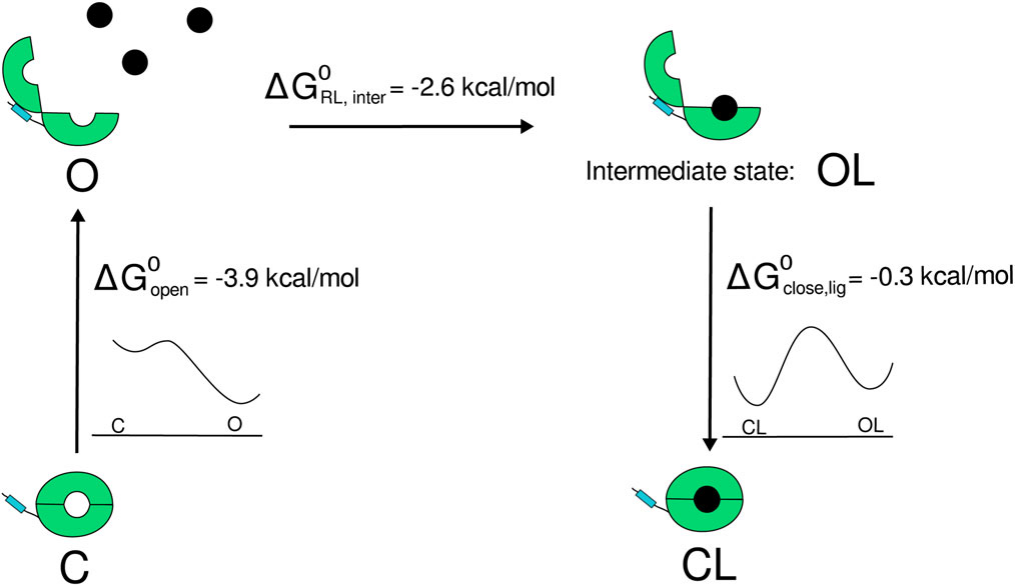
Ligand‐binding mechanisms and SBD2's conformational changes: The open protein form is locked via hydrophobic interactions between the subdomains in the C‐terminal region (highlighted by blue rectangles). SBD2 recognizes its substrate via a two‐step mechanism: GLN binds to the active site of the larger subdomain D1 of SBD2 in open conformation, forming an intermediate state. The ligand then induces a transition to the closed state by stabilizing the closed conformation of SBD2

Combined with the GLN binding in the unrestrained simulations it is possible to suggest a thermodynamic model for the binding and coupled conformational switching process (Figure 10). The total calculated binding free energy of ~3 kcal/mol suggests only weak binding in the mM range compared to a K_d_ in the μM regime measured experimentally.^10^, ^20^ In the absence of the GLN ligand the open form is favored and transition to the closed form involves a significant transition penalty and barrier. The GLN ligand has already significant affinity to the open form (binding only to the D1 domain). The calculated binding free energy for this step can only be considered as estimate because it was derived from unrestrained MD simulations with only few bound/unbound transitions. Surprisingly, the transition to the closed form also involves a free energy barrier (such that a spontaneous transition in the unrestrained simulations was not observed) and results only in a small further stabilization of the GLN‐bound form.

## 3 CONCLUSIONS

The SBD2 protein is an important subunit of the GlnPQ transporter system and binds specifically GLN molecules in order to deliver it to the translocation system of the GlnPQ transporter. It has been investigated extensively using biochemical experiments, NMR spectroscopy,^21^ X‐ray crystallography^10^ and time‐resolved smFRET experiments.^6^, ^19^, ^20^ The structural as well as smFRET studies demonstrate a global conformational transition coupled to ligand binding from an open apo to a closed domain arrangement upon ligand binding. The smFRET experiments indicate occasional domain closing events in the absence of GLN molecules that increase significantly in frequency upon addition of GLN ligands.^19^ In the present study our MD simulation results indicate that GLN ligands can bind to SBD2 site‐specifically with low affinity at exactly the ligand binding position found experimentally on just the D1 domain without an immediate domain closing motion. This indicates that ligand binding and domain motion do not occur simultaneously as expected in a classical induced fit binding process. However, HREUS simulations clearly indicate that a bound ligand strongly stabilizes the closed arrangement but the transition from an open to a closed domain state involves a significant energy barrier that is not crossed on the time scale of our unrestrained MD simulations. In smFRET experiments the binding of the GLN ligand itself cannot be detected and only an increased tendency of closing in the presence of GLN is observed.^19^ However, smFRET experiments with added L‐Arginine prevents GLN induced closing of SBD2.^20^ L‐Arginine binds to SBD2 and at sufficient concentration competes with GLN (hence binds at the same pocket of D1) but does not induce closing. This supports the present result that binding and closing can be separated. The present major indication of a pre‐equilibrium of GLN binding to just the D1 domain and only a small stabilizing contribution upon closing makes sense from a biological view point: GLN needs to be bound with modest affinity but for the transport it is important that the free energy cost of domain opening to release GLN is small to minimize the dissociation of the SBD2 complex before releasing the substrate to the ABC transporter.

Another major result of the present study is the observation that the D1‐tail_471‐484_ –D2‐helix_418‐427_ interaction is a decisive element to stabilize the open state and it controls the barrier for the open‐closed transition. This interaction element is typical for the SBD2 class of periplasmic ligand binding proteins and it is likely that it plays also in these cases a similar key role as in case of SBD2. The weakening or disruption of the contact (by the Leu480Ala substitution) resulted in rapid (barrier less) closing and it also destabilizes the open state such that occasionally closed states are sampled in the absence of ligand on the present MD timescale (experiments on the SBDLeu480Ala mutation show indeed an increased population of the closed‐form already in the absence of substrate, T. Cordes personal communication).

The barrier for the transition from the closed SDB2 state to the open state is found to be significant in case of a bound GLN but considerably smaller for SDB2 without a ligand. Hence, the prediction from this result is that the lifetime of the closed‐form should be longer in the presence of a ligand than without a ligand. However, smFRET studies show that the addition of GLN increases the frequency for open‐to‐close transitions.^19^ This agrees qualitatively with the simulation results because the barrier height in the absence of ligand is larger than in its presence in the binding cleft but the lifetime of the closed state was found to be similar both in the presence and absence of ligand.^19^ Such scenario predicts a similar barrier height for the global opening process in the presence and absence of ligand which was not observed in the simulations. It is possible that differences in the diffusivity along the open‐closing pathway in the presence or absence of GLN may play a role. However, it should be kept in mind that due to limited force field accuracy a quantitative agreement with all experimental results is not expected. Future simulation studies could give important insight into similarities and differences of the molecular substrate binding mechanism of related periplasmic binding proteins.

## 4 MATERIALS AND METHODS

### 4.1 *Unrestrained MD simulations*

The crystal structures of the open (PDB:4KR5) and the GLN bound closed SBD2 protein (PDB:4KQP) from *Lactococcus lactis* ^10^ served as start structures for the Molecular Dynamics (MD) simulations. The CUDA accelerated PMEMD^31^ version of the AMBER16 software package^32^ was used for all simulations employing the ff14SB force field parameters.^33^ The protein molecules were solvated in explicit TIP3P water^34^ in an octahedral box with a minimum distance of 10 Å to the box boundaries. The protonation state of the titratable amino acids were predicted via Poisson‐Boltzmann calculations, using the free open‐source web server H++.^35^ Simulations were started from the closed or open form in the absence or presence of GLN and a L480A mutation following the same equilibration protocol outlined below. In case of the MD simulations starting from the open apo SDB2 in the presence of substrate six GLN molecules were placed randomly into the simulation box resulting in a ligand concentration of ~25 mM. Sodium and chloride ions were added to neutralize the system and reach an ion concentration of 100 mM. After an energy minimization of 2000 steps of the steepest descent, the systems were gradually heated up to 300 K while keeping positional restraints (force constant of 10 kcal·mol^−1^·Å^−2^) on the protein non‐hydrogen atoms during 1 ns simulation time. The restraints were gradually removed for another 1 ns followed by unrestraint production simulations of more than 3 μs. All bonds involving hydrogens were kept at optimal length using Shake.^36^ The hydrogen mass reparation scheme was used allowing a time step of 4 fs.^36^ Trajectory analysis was performed using the cpptraj and pytraj tools^37^ of the AMBER16 suite. Visualization of structures and trajectories was performed using VMD.^38^

### 4.2 *Replica‐exchange umbrella sampling simulations*

Umbrella sampling (US) simulations coupled with Hamiltonian Replica exchanges (H‐REUS) between neighboring US intervals^39^, ^40^ were performed to obtain the potentials of mean force for the opening/closing transition of SBD2 with and without GLN present in the binding pocket. The distance between centers‐of‐mass of backbone atoms in two regions in the domains D1 and D2 of SBD2 served as a reaction coordinate ξ. The first center in the larger domain D1 contains the atoms CA, C, N of the residues 306–308, whereas the CA, C, N atoms of residues 396 and 397 form the other center‐of‐mass in D2. These segments are in close vicinity in the closed‐form (illustrated in Figure S6). A set of 16 US windows biased by harmonic potentials with force constants of 1 kcal·mol^−1^·Å^−2^ was generated with an equidistant spacing of 1 Å covering distances from 6 Å up to 21 Å. During the replica exchange process exchanges between neighboring US windows were attempted every 1 ps reaching an acceptance rate of 25–45%. The equilibrium distance for the reaction coordinate in the unrestrained simulations of closed and open SBD2 structure was 7.7 Å and 20 Å, respectively. H‐REUS simulations were carried out in the presence and absence of GLN in the binding pocket on the D1 domain. In each case one simulation was started from the open SBD2 configuration and the other starting from closed SBD2, resulting in a total of four H‐REUS simulations. The overall simulation time for the ligand‐bound state amounted to ~15.1 μs for all 16 US windows, where each US window was simulated for ~400 ns for the first simulation and ~ 540 ns for the second simulation, respectively. For the unbound state the total simulation time amounted to ~16.6 μs, where each US window was simulated for ~300 ns for the first simulation and ~ 741 ns for the second simulation. The simulated distributions along the reaction coordinates for the ligand‐bound and unbound state were analyzed by employing the weighted histogram analysis method (WHAM)^41^ yielding the corresponding free energy profile. Here, the implementation by Alan Grossfield^42^ was used which also allows error analysis via Monte Carlo Bootstrapping.

### 4.3 *Evaluation of trajectories using the MMPBSA technique*

Average interaction energy calculations on the unbiased trajectories have been conducted using the MMPBSA trajectory post processing method,^43^ utilizing the MMPBSA.py program of the Amber package.^44^ The purpose of the calculations was to obtain an estimate on the contribution of individual residues in the protein on the binding to the GLN ligand. Here, the “single trajectory approach” was used, assuming that there are no significant conformational changes upon binding and yielding the mean interaction between substrate and receptor decomposed for each residue at the binding region. In each case 5,000 frames in the ligand‐bound state of SBD2 sampled during free unrestrained MD simulation (Figure 2b) in the range from 1,000 up to 2,500 ns have been evaluated. The ion concentration was set to 100 mM (same as in the explicit solvent simulations). The harmonic conformational entropy contribution was included for calculating the total binding energy.

## AUTHOR CONTRIBUTIONS

Conceptualization, Martin Zacharias; Investigation, Maximilian Kienlein; Writing, Maximilian Kienlein and Martin Zacharias; Resources, Martin Zacharias, Supervision, Martin Zacharias, Funding Acquisition, Martin Zacharias.

## Supporting information


**Appendix** S1: Supporting information
