# Supplementary material for: Ligand binding and global adaptation of the GlnPQ substrate binding domain 2 revealed by molecular dynamics simulations
Source: Protein Sci. 2020 Nov 3;29(12):2482–94. doi: 10.1002/pro.3981 (PMC7679957; doi:10.1002/pro.3981)
Supplement: Supplementary file 1 — Appendix S1: Supporting information [file PRO-29-2482-s001.pdf]

Supporting Information:
Ligand binding and global adaptation of the GlnPQ substrate binding domain 2 revealed by Molecular Dynamics simulations

Maximilian Kienlein and Martin Zacharias

Physik-Department T38, Technische Universität München, James Franck Str. 1,
85747 Garching, Germany

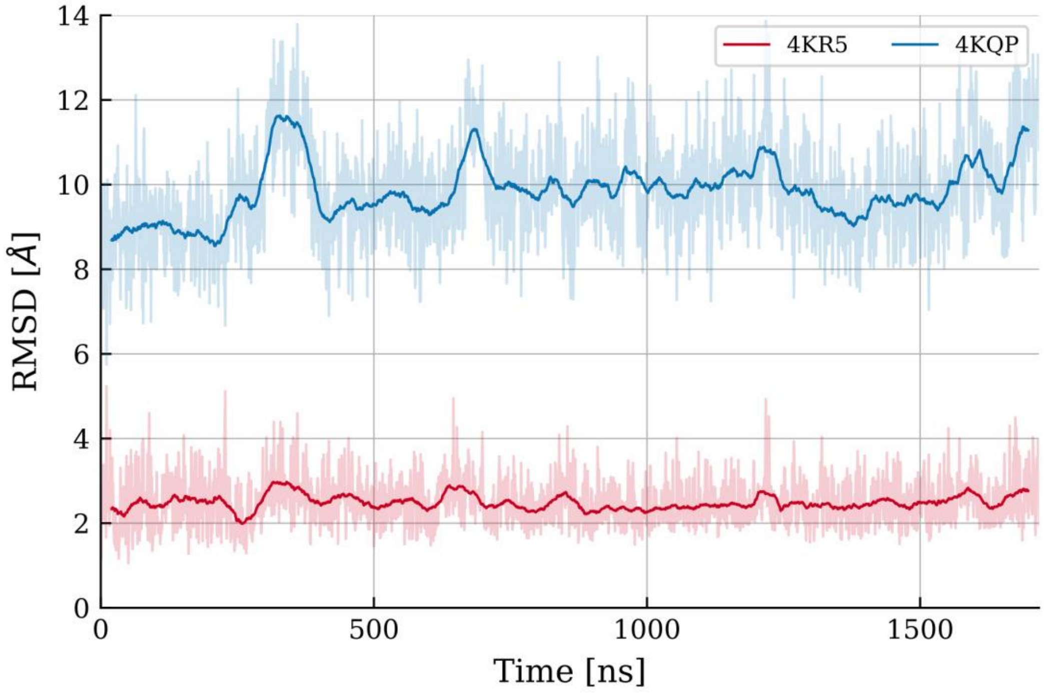

Figure S1: Root-mean-square deviation (RMSD) of the open state of SBD2 (in the absence of GLN) with respect to the crystal structures of open (PDB: 4KR5, red) and closed (PDB: 4KQP, blue) conformation. In the absence of L-glutamine (GLN) ligands the RMSD stayed in the range of ~3 Å with respect to the open crystal structure over 1.8 µs simulation time and no indication of spontaneous global domain closing.

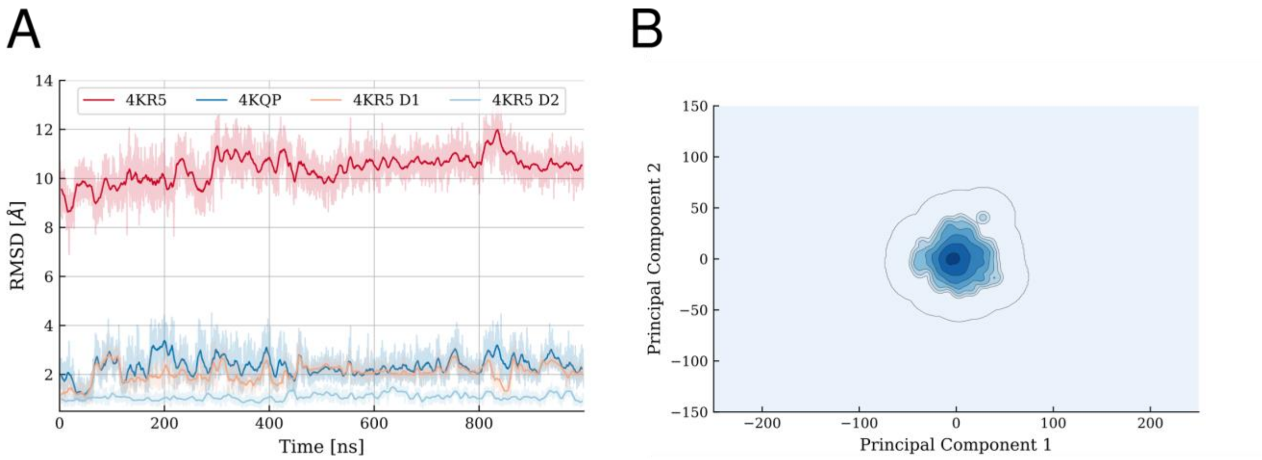

Figure S2: Free simulation of the closed, liganded SBD2 (with bound GLN). (A) Sampled RMSD values with respect to the crystal structures (open state: red; closed state: blue) and the two domains of the open conformation (D1: orange; D2: lightblue). (B) The same trajectory projected on to the first two eigenvectors. The protein remains closed, showing no major deviations from the crystal structure (PDB: 4KQP).

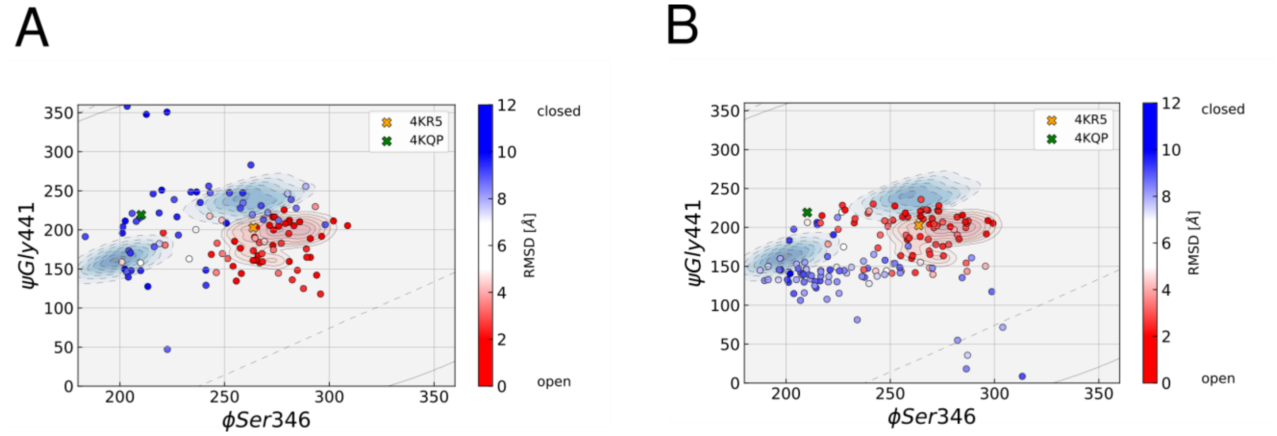

Figure S3: Conformational changes in the β-sheet hinge region upon the opening/closing of the in silico Leu480Ala mutated SBD2. Distinct Φ-SER346 and ψ-Gly441 angle distributions along the different transitions (A) Closing transition with the ligand GLN present in the binding pocket. (B) Both closing as well as opening in the simulation without ligand (see also Figure 8).

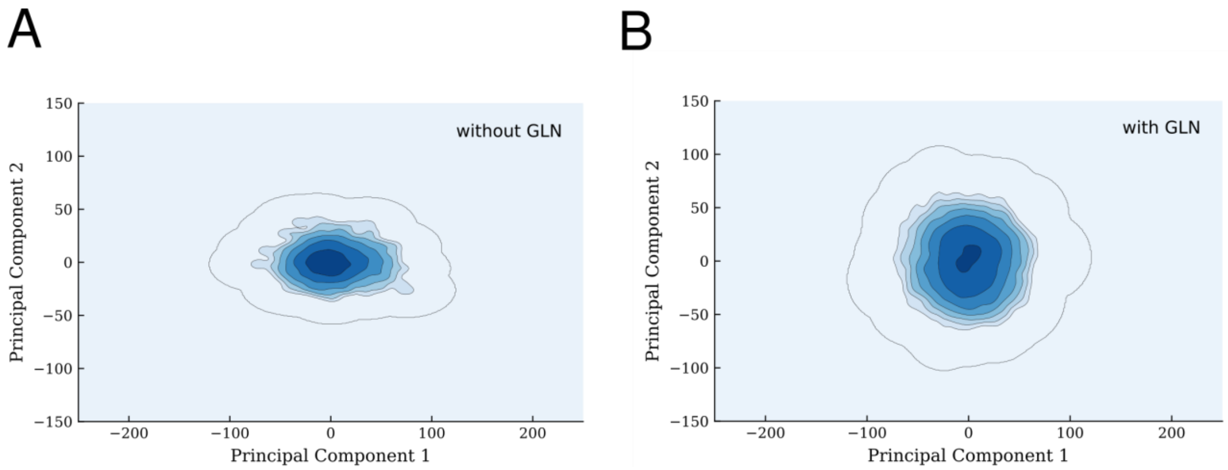

Figure S4: Principal Component Analysis of extensive unrestrained MD simulations of the open SBD2 state (PDB: 4KR5). (A) PCA of the open-unliganded SBD2 simulation (see Figure S2). (B) Addition of SBD2's specific ligand GLN does not trigger a global transition in the simulated timescales to the closed state (see also RMSD plots shown in Figure 2).

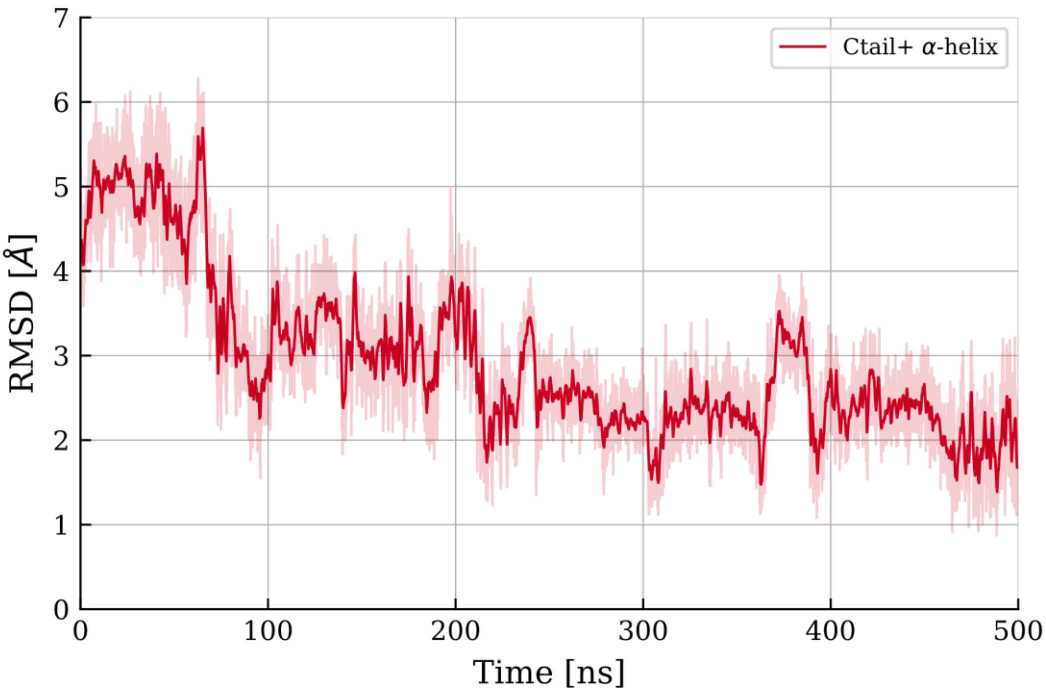

Figure S5: RMSD of C-terminal D1-helix$_{471\text{-}484}$ – D2-helix$_{418\text{-}427}$ regions with respect to the crystal structure of SBD2's open conformation upon the global opening transition (see Figure 4).

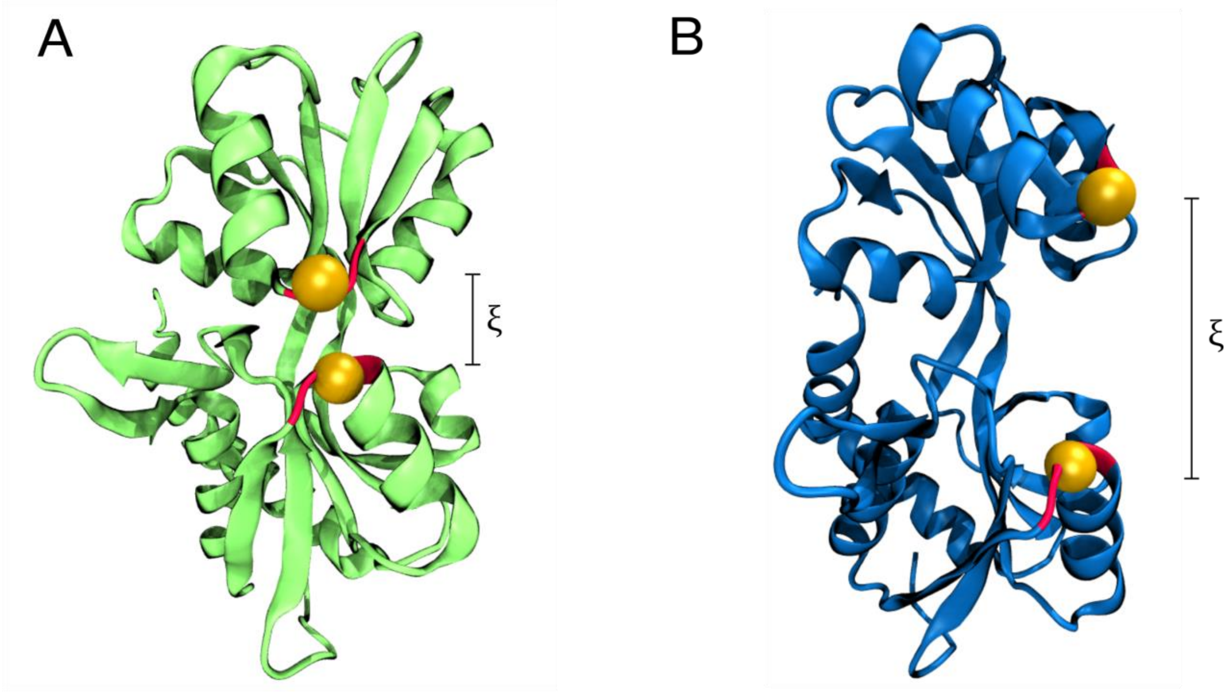

Figure S6: Illustration of the center-of-mass (yellow spheres) reaction coordinate ξ (backbone atoms of residues 306-308 of the D1 and residues 396 and 397 in D2, marked in red) for H-REUS simulations of the opening/closing transition of SBD2. (A) The crystallized structure of the closed ligand bound SBD2 (PDB: 4KQP) corresponds to a value of ~ 7.7 Å for the distance ξ between the two centers. (B) In the open conformation (PDB: 4KR5) ξ reaches values of ~ 20 Å.

Table S1: Crystal structures of several substrate binding domains, sharing a common D1/D2 composition, connected via antiparallel β-sheets. All of these SBD's form increased C-tail contacts to the D2 domain in the open (apo) conformation relative to the closed ligand-bound conformation.

Substrate Binding Domain	Apo conformation	Holo conformation
SBD2	4KR5	4KQP
OpuAC	3L6G	3L6H
PhnD	3S4U	3QUJ
MalE	1OMP	1ANF
FbpA	1SI1	1SIQ
